# From Dandruff to Deep-Sea Vents: *Malassezia*-like Fungi Are Ecologically Hyper-diverse

**DOI:** 10.1371/journal.ppat.1004277

**Published:** 2014-08-21

**Authors:** Anthony Amend

**Affiliations:** 1 Department of Botany, University of Hawaii at Manoa, Honolulu, Hawaii, United States of America; Duke University Medical Center, United States of America

## Introduction

As the dominant component of the mycobiota on human skin [1] —both healthy and diseased [2] —the genus *Malassezia* has received a fair amount of attention. Since the middle of the 19th century, researchers have linked these fungi with skin maladies such as dandruff and eczema [3], but their difficulty to culture axenically long hampered studies of their systematics and diversity [4]. *Malassezia* is the sole genus within the fungal order Malasseziales, contained within the proposed subphylum Malasseziomycetes (anonymous reviewer; personal communication). Although *Malassezia* is sister to the so-called “smut” plant pathogens, they are markedly divergent in ecological terms. A hallmark of *Malassezia* species is their incomplete fatty acids synthesis metabolic pathway, and reliance, instead, on a suite of extracellular lipases, phospholipases, and acid sphingomyelinases [5]. In fact, only a single species, *M. pachydermatis*, is able to survive in axenic culture lacking lipid amendment [6].

Until recently, it was assumed that *Malassezia* evolved into a specialized and narrow niche associated with the skin of mammalian hosts. However, culture-independent studies of fungi from environmental samples show that *Malassezia* are exceedingly widespread and ecologically diverse [3]. Recent studies in little-characterized marine environments point to extensive diversification of *Malassezia*-like organisms, providing exciting opportunities to explore the ecology, evolution and diversity of this enigmatic group.

## What Do We Know about the Diversity and Distribution of Putative *Malassezia* spp. from Environmental Sequences?

Despite being difficult to cultivate, putative *Malassezia* are readily detected in environmental DNA samples using standard fungal “barcoding” approaches. Scanning GenBank and the scientific literature, therefore, is useful for approximating occurrence patterns. DNA sequences identical to *M. globosa* and *M. restricta*, which are both well characterized as human skin associates, appear to be cosmopolitan. *M. restricta* may be particularly widespread, and DNA sequences similar to these species have been detected in habitats as diverse as deep-sea sediments [7], hydrothermal vents [8], stony corals [9], lobster larval guts [10], Japanese Eel (*Anguilla japonica*) gut and muscle tissue [11], Antarctic soils [12], [13], on the exoskeleton of soil nematodes [14], and various plant roots including mycoheterotrophic species such as orchids (e.g., [15]). Remarkably, the ribosomal DNA sequences of *Malassezia* in these studies are nearly identical to those of human associates, suggesting either a very recent divergence in habitat or else that these organisms are highly tolerant to some of the planet's most extreme environments. Unsurprisingly, *Malassezia* sequences are not uncommon in studies of human dwellings [16], where human skin contributes substantially to house dust.

Both putatively familiar and novel *Malassezia*-like organisms are abundant on living marine hosts. Pollock and colleagues [17] report *Malassezia* dermatitis in captive pinnipeds. Two recent studies of marine biotrophic fungi show that *Malassezia*-like organisms can numerically dominate fungal communities on invertebrates. A cultivation-independent study of marine sponges from Hawaii [18] revealed a high diversity of *Malassezia*-like sequences, and indicated that a subset of these differed from the adjacent water column. The authors' analysis further suggested that some of these putative *Malassezia* taxa are host specific at the species level. In a study of the scleractinian coral *Acropora hyacinthus*, Amend and colleagues [9] found that a phylogenetically diverse suite of *Malassezia*-like DNA sequences comprised the majority of fungi on apparently healthy colonies. A single taxon, most closely resembling *Malassezia globosa*, was significantly more abundant amongst corals located in warmer water.

## Are Marine *Malassezia* Related to Terrestrial Species?

**Figure 1:**
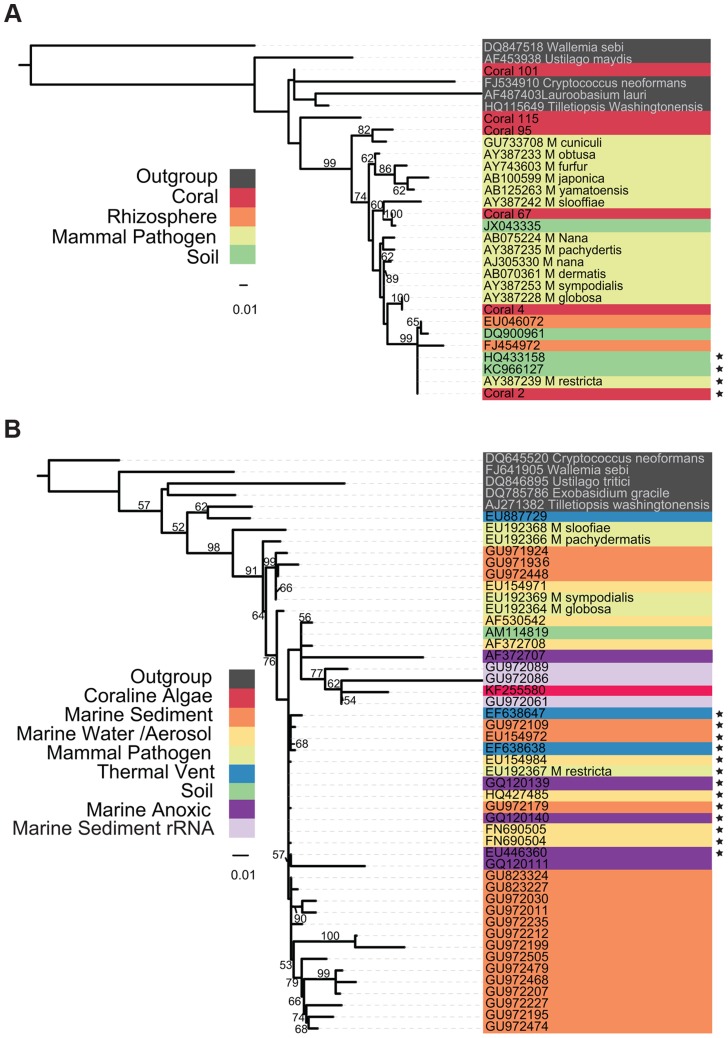
Phylogenetic Tree of *Malassezia*-like sequences derived from environmental DNA sequences and isolates. Tree topology was inferred using the best of 1,000 bootstrap replicates of a Maximum Likelihood tree building algorithm (RAxML). Bootstrap support values >50% are indicated. Organism origin is indicated by highlight coloring. Sequences with 99% or greater identity to *M. restricta* are starred. (A) Tree containing sequences from the D1 and D2 variable domains of the large ribosomal subunit. Coral sequences are deposited in the NCBI SRA, accession #SRP005168. (B) Tree containing sequences spanning the V4 hypervariable region of the ribosomal small subunit; most sequences were compiled in [19].

The evolutionary origins of marine *Malassezia* and their relatedness to better- characterized terrestrial species is a matter of speculation. A phylogeny compiled from environmental samples and sequenced isolates (Figure 1) demonstrates a tremendous amount of phylogenetic novelty contained within and adjacent to the *Malassezia* lineage. Evidence from both large and small subunit loci of the ribosomal cistron demonstrate well-supported clades from various environments, including a large monophyletic group of marine water column mycoplankton, sequences from separate studies of marine anoxic environments, and combinations of host-associated (coral and coralline algae) *Malassezia* that group with presumably free-living taxa in various marine and terrestrial habitats. The relatively long branch lengths separating some of these isolates from their sister taxa suggest either a particularly rapid diversification, or, alternatively, that intermediate taxa remain to be sampled and sequenced.

A phylogeny of environmental sequences from putative *Malassezia* shows that marine taxa are interdigitated amongst those from human hosts and other terrestrial substrates and do not form a single monophyletic clade. This topology suggests repeated transitions between marine and terrestrial habitats, a pattern not atypical of marine fungi within other Dikarya lineages, including yeasts [19]. The relative ease with which fungi transition between such different environments may owe to the strength of chitinous cell walls, which withstands increased water and osmotic pressures in deep saline habitats [19].

## What Can We Infer about the Ecology and Trophic Status of Marine *Malassezia*?

The tremendous diversity of habitats in which *Malassezia*-like organisms are found suggests that marine species of this group may incorporate a spectrum of trophic strategies ranging from saprotrophy to biotrophy. Resident *Malassezia*-like organisms on seemingly healthy coral and sponge hosts may be commensals, latent pathogens awaiting host immunosuppression, or both, depending on host and environmental context. A study of crustose coralline algae around Palmyra Atoll found that a *Malassezia* phylotype was abundant in banding disease lesions [20]. Incidence of the disease increased by an order of magnitude following an el Niño event. A laboratory manipulation study showed that disease virulence correlated with an interaction between increases in CO_2_ and temperature. Despite efforts, the authors were unable to cultivate the fungus, and it remains to be tested if *Malassezia* is the cause or merely a symptom of the banding disease. Nevertheless, the study presents the possibility that a putative *Malassezia* may act as a pathogen in nonmammal hosts under certain environmental contexts. The high incidence and virulence of the disease raises the possibility that when combined with environmental perturbations, marine *Malassezia* may even exert bottom-up control on reef community structure.

## How Do We Know that *Malassezia* Detected in Marine Environmental DNA Aren't Contaminants?

Given the high incidence of *Malassezia* species on human skin [1], [2], a healthy skepticism is warranted since mammalian skin cells from terrestrial sources could potentially accumulate in marine samples, or contamination by lab personnel could result in false positives. Potential for contamination is particularly high when environmental DNA sequences are generated using sensitive, high-throughput methods. Nevertheless, multiple lines of evidence support the position of *Malassezia*-like organisms as true marine residents. Edgcomb and colleagues [21] reported a high proportion of *Malassezia*-like sequences in deep-sea sediments detected by sequencing environmental RNA. Because single stranded RNA degrades quickly in situ, its presence supports the notion of active growth as opposed to DNA “contamination” in this habitat. Furthermore, the RNA sequences were distinct from those of any organism known to associate with mammalian hosts, excluding the possibility of lab contamination. A follow-up study using even more stringent protocols and negative controls to exclude exogenous nucleic acids detected *Malassezia*-like sequences in samples located at depths of 1.6 and 45.1 meters below the sea floor [22]. Fungal community composition overall was highly correlated with site geochemistry, suggesting the environmental selection of a metabolically active assemblage. Similarly, an analysis of actively transcribed genes (mRNA) from a coral habitat identified components of multiple metabolic pathways allied with sequenced *Malassezia* genomes [9] —further evidence that these fungi are alive and metabolically active underwater. The fact that *Malassezia*-like organisms are frequently found in remote marine locations far from humans (e.g., [7], [8], [21], [23]–[27], and many others) also renders the terrestrial input hypothesis less likely.

## What Are Future Directions for Research into Marine *Malassezia*?

The remarkable environmental plasticity of *M. restricta* lends itself to population-level studies of adaptation and acclimatization among the Earth's most extreme environments. How do differences in gene content and transcription correlate with residence in arctic soils versus deep-sea vents? What traits mark the transition from saprobic to pathogenic lifestyles? How many times has a marine (or terrestrial) lifestyle evolved independently?

As a model system, the genus *Malassezia* has much to offer: three sequenced genomes, *M. globosa*, *M. restricta*, and *M. sympodialis*, contain fewer than 9 Mb and 5,000 genes [28], [29], placing them amongst the smallest free-living genomes in the kingdom Fungi. Furthermore, although the sexual cycle has not been observed in this group, a genomic signature of bipolar mating exists [3], [5], [29], [30]. The genus *Malassezia* contains a rich and potentially novel suite of enzymes and metabolites [2] from a variety of inhospitable and relatively unexplored habitats.

Arguably, the greatest challenge to studying marine *Malassezia* is in obtaining axenic cultures, and no marine isolates, to my knowledge, have been recovered to date. Even among species associated with the better-characterized terrestrial mammalian hosts, lab cultivation can be a hit-or-miss affair, involving media that requires a variety of specialized and exotic fatty acids [2], [3], [6]. Studying marine environments introduces additional complexities, such as requiring specialized pressure and salinity growth conditions,that can further complicate cultivation efforts.

Nevertheless, there is much to learn about the basic biology and physiology of host-associated marine *Malassezia*-like organisms independent of culturing. Some of these questions can be addressed using microscopy in conjunction with labeling techniques such as fluorescence in situ hybridization (FISH). Even basic questions about where *Malassezia*-like organisms reside on corals remains to be answered. Are *Malassezia*-like organisms associated with coral mucus, for example, or are they more closely associated with the dinoflagellate symbionts? Does their exclusion affect host fitness? Is there evidence of host specificity or co-evolution?

## Conclusions

Analysis of environmental sequences demonstrates that putative members of the *Malassezia* lineage likely rank among the most widespread fungi on the planet. They are found in a startling diversity of habitats and locations, from polar regions to deep-sea vents. *Malassezia*-like species appear to dominate certain marine habitats, which should most certainly be the focus of future research into the diversity and distribution of this enigmatic group. Clearly, considering *Malassezia* a mere epidermis-commensal is a definition that is only skin deep.
